# Mapping of Rice High-Tillering Dwarf Gene *HTD8* and Preliminary Functional Analysis of Its Involvement in Responses to Salt Stress and PEG-Induced Osmotic Stress

**DOI:** 10.3390/plants15213335

**Published:** 2026-10-28

**Authors:** Jun Liu, Xiaoyan Liu, Chenglei Zhong, Qianyun Jin, Yuhan Xue, Chenqi Jiang, Mei Lu, Yuchun Rao

**Affiliations:** College of Life Sciences, Zhejiang Normal University, Jinhua 321004, China; 18267889248@163.com (J.L.); 15058772853@163.com (X.L.); 15058406329@163.com (C.Z.); 15168158070@163.com (Q.J.); 15869166933@163.com (Y.X.); 19730384231@163.com (C.J.); lumei@zjnu.cn (M.L.)

**Keywords:** rice, high-tillering dwarf, plant hormone response, abiotic stress

## Abstract

Rice (*Oryza sativa* L.) is an essential staple crop worldwide, and tillering acts as a key target for molecular breeding because of its critical contribution to grain yield. In this study, a high-tillering dwarf mutant designated *htd8* was isolated from an ethyl methanesulfonate (EMS)-mutagenized population of indica rice cultivar ‘Shuangkezao (SKZ)’. The mutant exhibits reduced plant height, dramatically increased tiller number, shortened and yellow-chlorotic leaves. Compared with the wild-type (WT), *htd8* displays significant decreases in plant height, internode length, panicle branch number and 1000-grain weight, accompanied by a remarkable increase in tiller count. Genetic analysis demonstrated that the *htd8* phenotype is governed by a single recessive nuclear gene. By bulked segregant analysis (BSA) sequencing combined with fine mapping, the candidate gene was delimited to a 0.81 Mb genomic interval on rice chromosome 11, where two known tillering-related genes *D27* and *ZEBRA2* were excluded. Exogenous hormone assays revealed that *htd8* had decreased sensitivity to exogenous GA_3_ but enhanced sensitivity to the strigolactone analog GR24, and both GA_3_ and GR24 could repress tiller outgrowth in the mutant. Under salt stress and PEG-simulated osmotic stress, *htd8* achieved a notably higher survival rate relative to WT. The mutant accumulated more proline (Pro), maintained lower malondialdehyde (MDA) levels, and showed elevated leaf abscisic acid (ABA) content together with the upregulated expression of ABA biosynthetic-related genes. Transcriptome profiling of tiller buds indicated that *HTD8* modulates rice tillering and plant architecture by coordinating brassinosteroid, auxin, gibberellin and strigolactone signaling pathways, as well as carbon metabolism redistribution. This work provides theoretical insights into the molecular mechanism underlying high-tillering dwarfism and supplies valuable genetic resources for high-yield and stress-tolerant rice molecular breeding.

## 1. Introduction

Rice (*Oryza sativa* L.) is one of the most important food crops worldwide, and its plant architecture is a key agronomic trait determining yield [1]. Tillering number, plant height, leaf morphology, and grain size are critical components of an ideal plant type, ultimately determining yield potential by directly influencing the effective panicle number, grains per panicle, and thousand-grain weight [2,3]. Among these, the tiller number directly determines the effective panicle number and is one of the most significant traits affecting the yield in plant architecture regulation. Tillers originate from axillary buds located on the unelongated internodes at the base of leaves [4], and their development involves two stages: initiation and outgrowth of tiller buds. This process is precisely regulated by a complex interplay of genetic networks, plant hormones, and environmental signals, serving as a crucial node for balancing vegetative and reproductive growth and coordinating source–sink relationships [5].

The development of rice tillers is tightly controlled by multi-layered hormonal signaling and transcriptional regulatory networks. At the genetic level, tiller bud formation and elongation result from coordinated actions of multiple regulatory modules, including maintenance of shoot apical meristems, axillary bud initiation, and tiller bud formation. Axillary meristem initiation is a pivotal step in tiller development; *OsMOC1* encodes a core GRAS-type transcription factor GAI-RGA-and-SCR(GRAS) involved in axillary bud initiation. Loss in function of *OsMOC1* leads to nearly complete absence of tillers and a dramatic reduction in yield [6]. The stability of the *OsMOC1* protein is regulated by APC/C ubiquitin ligase components *OsTAD1*/*OsTE*, which target it for degradation via the ubiquitin–proteasome pathway, thereby finely tuning tiller number [7,8]. *OsMOC3* (*OsTAB1*) is a homolog of the Arabidopsis *WUSCHEL* (*WUS*) gene and interacts with *OsMOC1* to jointly activate downstream *OsFON1*, maintaining stem cell activity in axillary meristems [9]. *OsMOC3* upregulates the expression of *OSH1* and *OsWOX4*, ensuring proper establishment of axillary meristems.

Recent studies have shown that a complex network of hormones, including strigolactones (SLs), gibberellins (GAs), auxin (IAA), brassinosteroids (BRs), cytokinins (CKs), and abscisic acid (ABA), precisely regulates rice tillering and plant height [10]. Strigolactone acts as the central hormone suppressing tiller formation and forms the core hormonal regulatory layer inhibiting tiller elongation. SL is synthesized by enzymes encoded by genes such as *D10*, *D17*, and *D27*. After being recognized by the receptor *OsD14*, SL forms a protein complex with the F-box protein *OsD3* and the signal repressor *OsD53*, promoting *OsD53* ubiquitination and subsequent degradation via the 26S proteasome. This releases *OsD53*’s repression on the transcription factor *OsIPA1* (*OsSPL14*) [5]. Downstream of the SL signal lies the central architectural regulator *OsIPA1*/*OsSPL14. OsIPA1* and *OsD53* form a feedback loop and further activate downstream target genes such as *OsTB1* and *OsDEP1*, reshaping rice plant architecture through the modulation of gene expression [11]. In addition to promoting stem elongation, GA also suppresses tillering by regulating the homeostasis of key proteins. The DELLA protein *OsSLR1* is a central component of the GA signaling pathway and stabilizes the positive tiller regulator *OsMOC1*, promoting axillary bud formation. When GA binds to its receptor *OsGID1*, it triggers the degradation of *OsSLR1*, leading to reduced levels of the *OsMOC1* protein and suppression of tiller bud formation. In addition, high concentrations of GA can activate the APC/C^TAD1^ ubiquitin complex, accelerating the degradation of *OsMOC1* within axillary meristems and thereby restricting tiller formation, thus balancing stem elongation with tiller development [12,13]. ABA, as a stress-related hormone, also negatively regulates rice tillering. *OsTB1* directly binds to and activates *OsGT1* transcription; *OsGT1* further upregulates *OsNCED1*, a key gene in ABA biosynthesis, increasing endogenous ABA levels and suppressing tiller formation [14]. IAA maintains apical dominance by polar transport that inhibits axillary bud activation, whereas brassinosteroids positively promote tillering [12]. With advances in molecular genetics, several key genes regulating tillering and plant height, such as *MOC1*, *MOC3*, *LAX1*, *HTD1*, *D10*, *D14*, and *D27* have been cloned. However, the regulatory network controlling plant architecture still contains numerous uncharacterized loci and molecular mechanisms, particularly the coordinated regulation among hormonal signaling, chloroplast development, stress resistance, and tillering, which remains to be thoroughly elucidated [15].

SL and ABA both originate from the carotenoid biosynthetic pathway and share a common precursor, all-trans-β-carotene. Their functional connections extend beyond the regulation of tillering [16]. ABA is a central hormone in plant responses to abiotic stresses, mediating stomatal closure, accumulation of osmoprotectants, and transcriptional activation of stress-responsive genes under drought and salinity conditions [17]. Meanwhile, the crosstalk between SL and GA pathways regulates branching and root architecture [18]. This suggests that genes involved in plant architecture may simultaneously participate in stress response pathways, and multiple studies have provided experimental evidence supporting this notion. The ideal plant architecture gene *IPA1*/*OsSPL14*, a target of microRNA156, regulates panicle branching, suppresses ineffective tillers, and enhances stem mechanical strength. Wang et al. demonstrated that *OsIPA1* directly interacts with the salicylic acid receptor *OsNPR1* in the nucleus, preventing its degradation mediated by the E3 ubiquitin ligase *OsCUL3a*, thereby maintaining protein stability and synergistically activating downstream defense genes, significantly enhancing resistance to bacterial blight and blast disease in rice [19]. Li et al. identified *SDR_1_*, a semi-dwarf gene encoding a RING-H2-type E3 ubiquitin ligase, which targets EUI_1_, a key enzyme in GA inactivation, and mediates its degradation via the ubiquitin–proteasome system, precisely regulating endogenous GA homeostasis. In *sdr1* loss-of-function mutants, reduced endogenous GA levels lead to semi-dwarfism and lodging resistance, along with improved salt tolerance and resistance to bacterial blight during both seedling and entire growth stages, achieving coordinated improvements in dwarfism, lodging resistance, and multiple stress tolerances [20]. Furthermore, the gain-of-function mutant *tld1-D* of *TLD1* (*OsGH_3.13_*) exhibits increased tiller number, reduced plant height, and significantly enhanced drought tolerance [21]. Nevertheless, multifunctional genes capable of simultaneously regulating plant height, tillering, leaf development, and non-biological stress responses remain relatively rare, and their underlying molecular regulatory networks require further investigation.

Wang et al. found that the beneficial allele *HTD1^HZ^* of the SL biosynthesis gene *HTD1* is a major contributor to the high-tillering and high-yielding traits in Huazhan, promoting the tiller number without compromising grain setting rate, thereby increasing the yield, providing important insights for breeding high-yield, multi-tiller rice varieties [22]. This study focused on the multi-tillering dwarf mutant *htd8* (*high-tillering dwarf 8*), conducting phenotypic characterization, agronomic trait analysis, ultrastructural observations of leaves and chloroplasts, exogenous hormone response assays, PEG-induced osmotic stress resistance evaluation, gene mapping, and tiller bud transcriptome analysis. The aim was to clarify the molecular pathway by which *HTD8* regulates tillering and plant height, as well as its role in PEG-induced osmotic responses, thereby providing new genetic resources and theoretical support for molecular breeding of ideal rice plant architecture and stress tolerance improvement.

## 2. Results

### 2.1. Stem Mechanical Strength and Cell Wall Component Analysis

**Figure 1:**
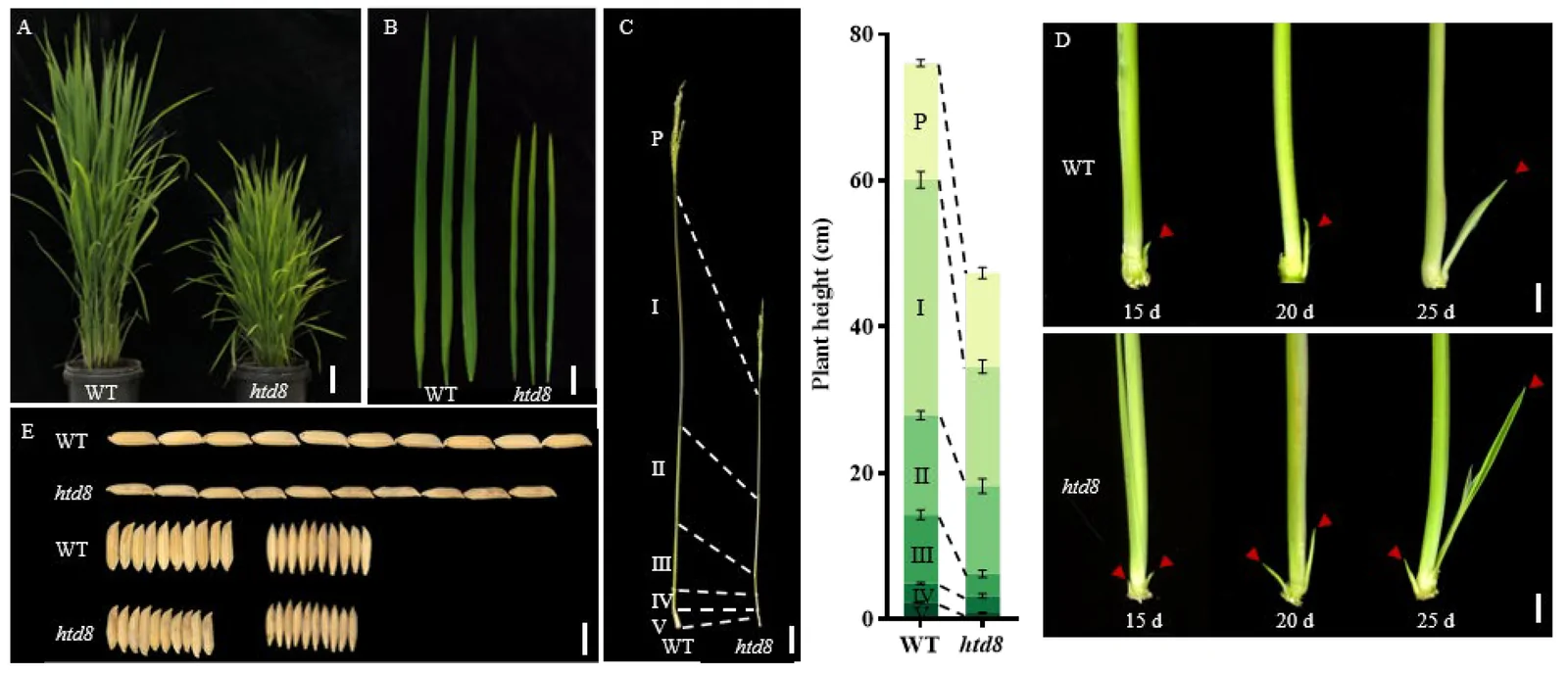
Phenotypic comparison between WT and *htd8* mutant rice. (**A**) Phenotypes of WT and *htd8* at tillering stage, scale bar = 10 cm. (**B**) Leaf phenotypes of WT and *htd8* at tillering stage, scale bar = 2 cm. (**C**) Comparison of internode lengths between the main culm of WT and *htd8*, scale bar = 10 cm. P, I, II, III, IV, and V represent inflorescence, first, second, third, fourth, and fifth internodes, respectively. (**D**) Morphology of tiller buds at three-leaf stage in WT and *htd8*: red arrowheads mark tiller buds, scale bar = 1 mm. (**E**) Grain phenotypes of WT and *htd8*, scale bar = 1 cm.

During plant development and maturation, we systematically examined key agronomic traits of the WT and the *htd8* mutant (Figure 1 and Table 1). Compared to the WT, the first and third internodes of the *htd8* mutant were significantly shorter, resulting in a marked reduction in plant height. From 15 to 25 days after sowing, the *htd8* mutant exhibited a greater number of initial tiller buds and a faster growth rate than the WT, leading to a significant increase in tiller number, which reached approximately three times that of the WT at maturity. Additionally, the leaves of the *htd8* mutant were generally smaller and narrower, with obvious chlorosis at the leaf tips. The grain length, width, and thickness were reduced, and both primary and secondary panicle branches decreased in number, causing a significantly lower thousand-grain weight compared to the that of the WT.

### 2.2. Observation of Leaf Surface and Chloroplasts in the htd8 Mutant

**Figure 2:**
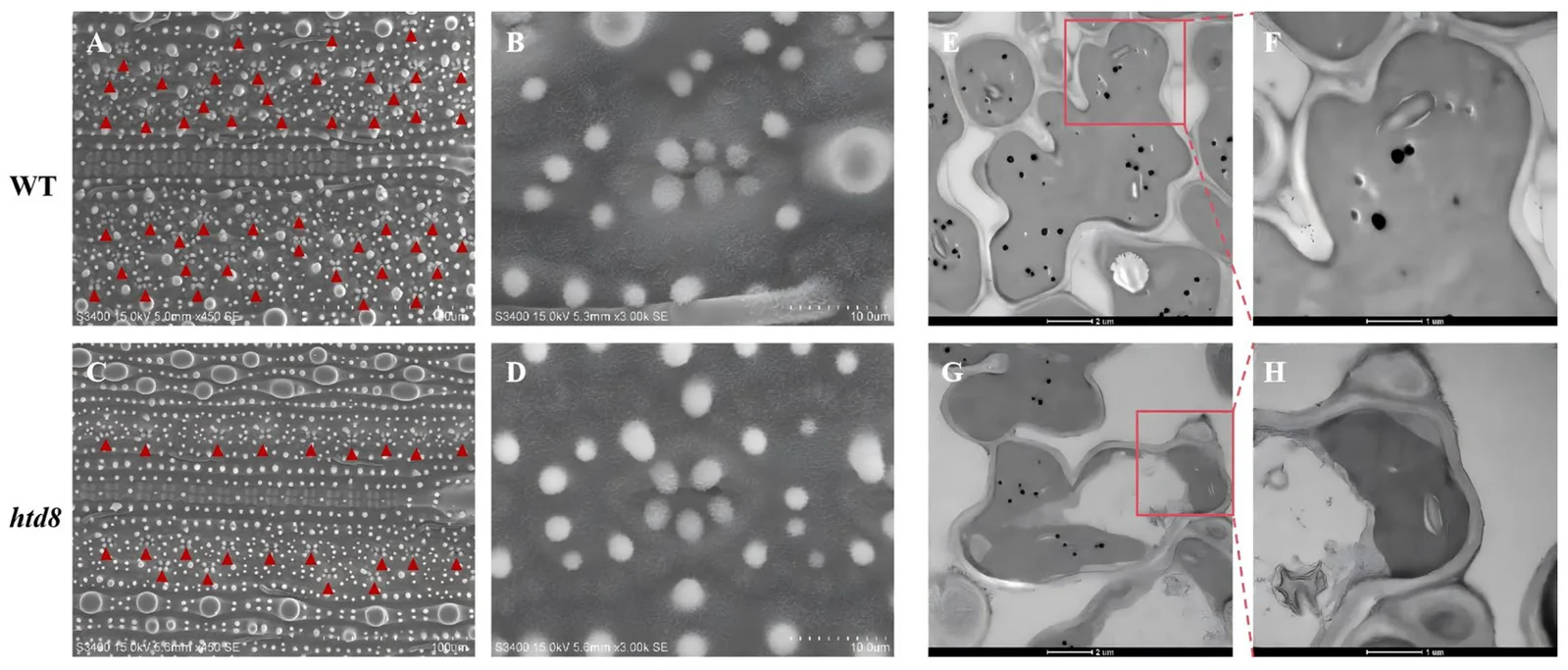
Comparison of leaf ultrastructure between WT and *htd8* rice. (**A**,**B**) SEM images of WT leaves at ×500 and ×3000 magnification, with scales of 100 μm and 10 μm, respectively. (**C**,**D**) SEM images of *htd8* leaves ×500 and ×3000 magnification, with scales of 100 μm and 10 μm, respectively; red arrowheads indicate stomata. (**E**,**F**) Chloroplast ultrastructure in WT leaves, with scales of 20 μm and 1 μm, respectively. (**G**,**H**) Chloroplast ultrastructure in *htd8* leaves, with scales of 20 μm and 1 μm, respectively.

Leaves of WT and mutant plants at the peak tillering stage were observed by a scanning electron microscope (SEM). As shown in Figure 2A–D, stomatal density and total number of stomata per unit area were higher in the WT than in the mutant, whereas the density of silicified protrusions was lower. TEM (transmission electron microscopy, TEM) revealed that in the mutant, chloroplasts were smaller in volume, irregularly shaped, and exhibited tightly disorganized thylakoid membrane structures (Figure 2E–H).

**Figure 3:**
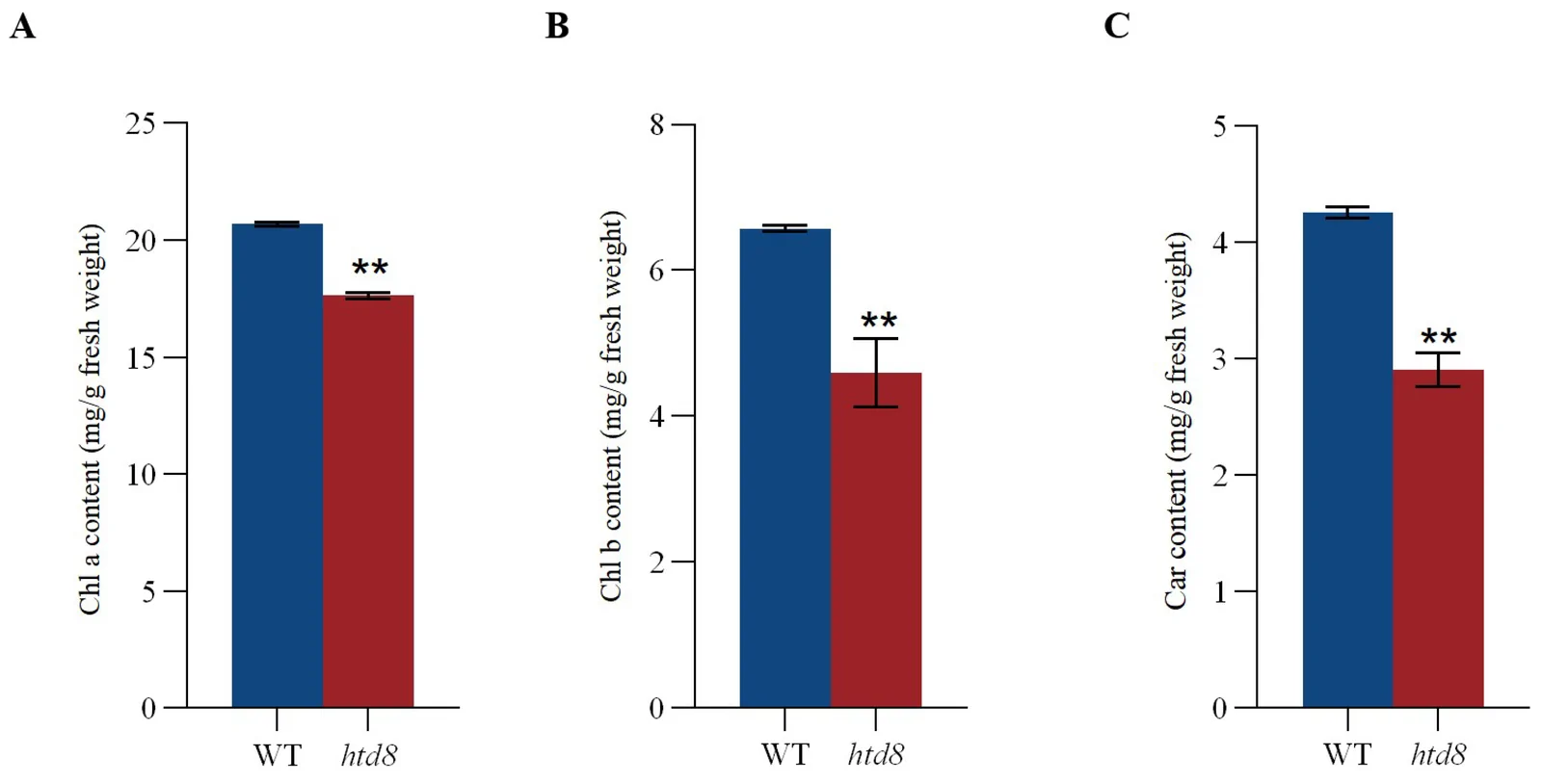
Results of photosynthetic pigment content measurements in WT and *htd8* mutant. (**A**) Chlorophyll a content in WT and *htd8* mutant. (**B**) Chlorophyll b content in WT and *htd8* mutant. (**C**) Carotenoid content in WT and *htd8* mutant. ** p < 0.01.

Photosynthetic pigment analysis revealed that the contents of chlorophyll a (Chl a), chlorophyll b (Chl b), and carotenoids (Car) in *htd8* leaves were significantly lower than those in the WT (Figure 3). Consequently, the *HTD8* mutation leads to a reduction in leaf stomatal density, thereby weakening transpiration. Simultaneously, chloroplast structural integrity is compromised, photosynthetic pigment levels decline, and photosynthesis was inhibited, thereby impairing rice leaf growth and development.

### 2.3. Effects of Exogenous Hormones on the Growth and Development of htd8 Mutant Seedlings

**Figure 4:**
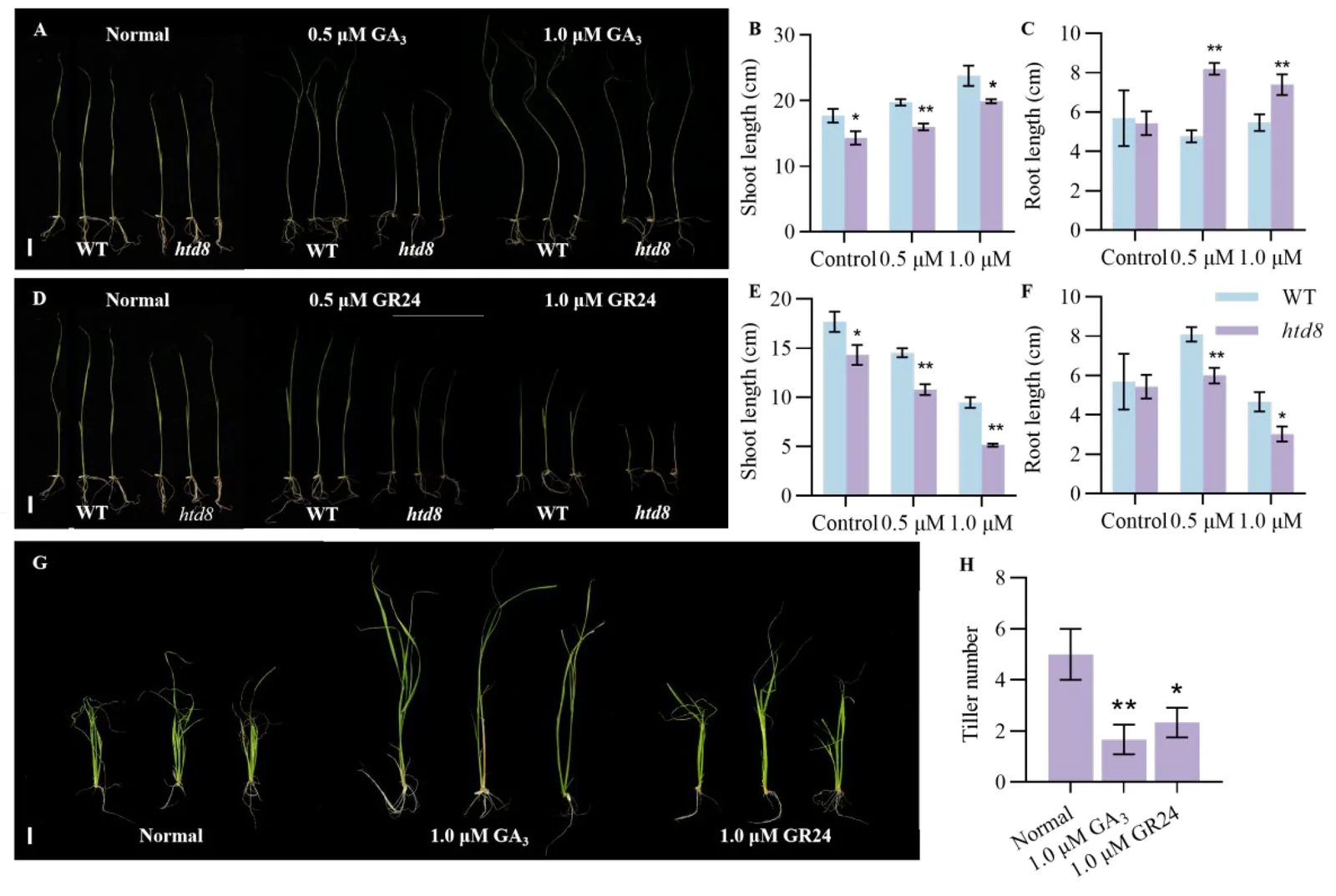
Phenotypes of WT and *htd8* mutant after exogenous hormone treatment. (**A**–**C**) Phenotypes of WT and *htd8* mutant following GA_3_ treatment. Bar = 1 cm. (**D**–**F**) Phenotypes of WT and *htd8* mutant following GR24 treatment. Bar = 1 cm. (**G**,**H**) Tillering status of WT and *htd8* mutant after treatment with GA_3_ and GR24. Bar = 2 cm. * *p* < 0.05; ** *p* < 0.01.

To investigate the effects of GA_3_ and SL on the growth and tiller number of the mutant, WT and *htd8* mutant seeds were cultured on 1/2 MS medium supplemented with different concentrations of exogenous hormones. Seven days after germination, plant height and root length were measured. As shown in Figure 4, compared to the WT, the *htd8* mutant showed insensitivity to GA_3_ in shoot growth, whereas its root length was significantly longer than that of the WT under GA_3_ treatment. The inhibitory effect of exogenous GR24 on rice seedling shoot growth increased with higher concentrations, and the *htd8* mutant exhibited significantly greater sensitivity than the WT. Meanwhile, GR24 promoted root elongation in the WT but inhibited root growth in the *htd8* mutant. Application of either GA_3_ or GR24 suppressed tiller formation in the *htd8* mutant. These results indicate that the *htd8* mutation alters the response to both gibberellin and strigolactone.

### 2.4. Response of the htd8 Mutant to Abiotic Stress

**Figure 5:**
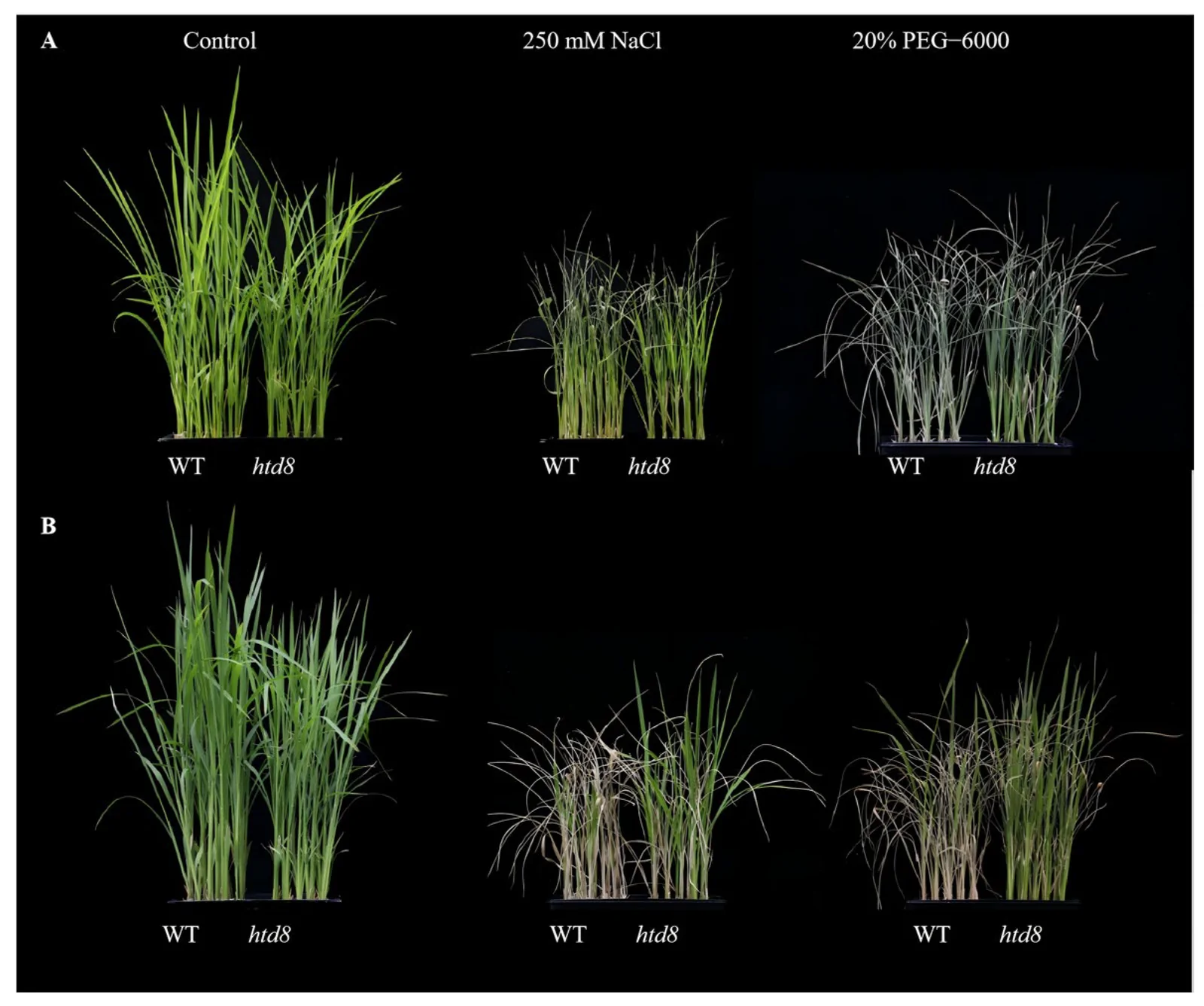
WT and mutant *htd8* seedlings under salt and PEG-induced osmotic stress. (**A**) Phenotype after abiotic stress. Bar = 2 cm. (**B**) Phenotype after stress recovery. Bar = 2 cm.

We further examined the survival rate and related parameters of the *htd8* mutant under salt stress (250 mM NaCl for 5 d) and PEG-induced osmotic stress (20% PEG-6000 for 7 d). As shown in Figure 5, the *htd8* mutant exhibited a higher survival rate than the WT after both salt and PEG-induced osmotic treatments, showing stronger resistance particularly under PEG-induced osmotic stress.

**Figure 6:**
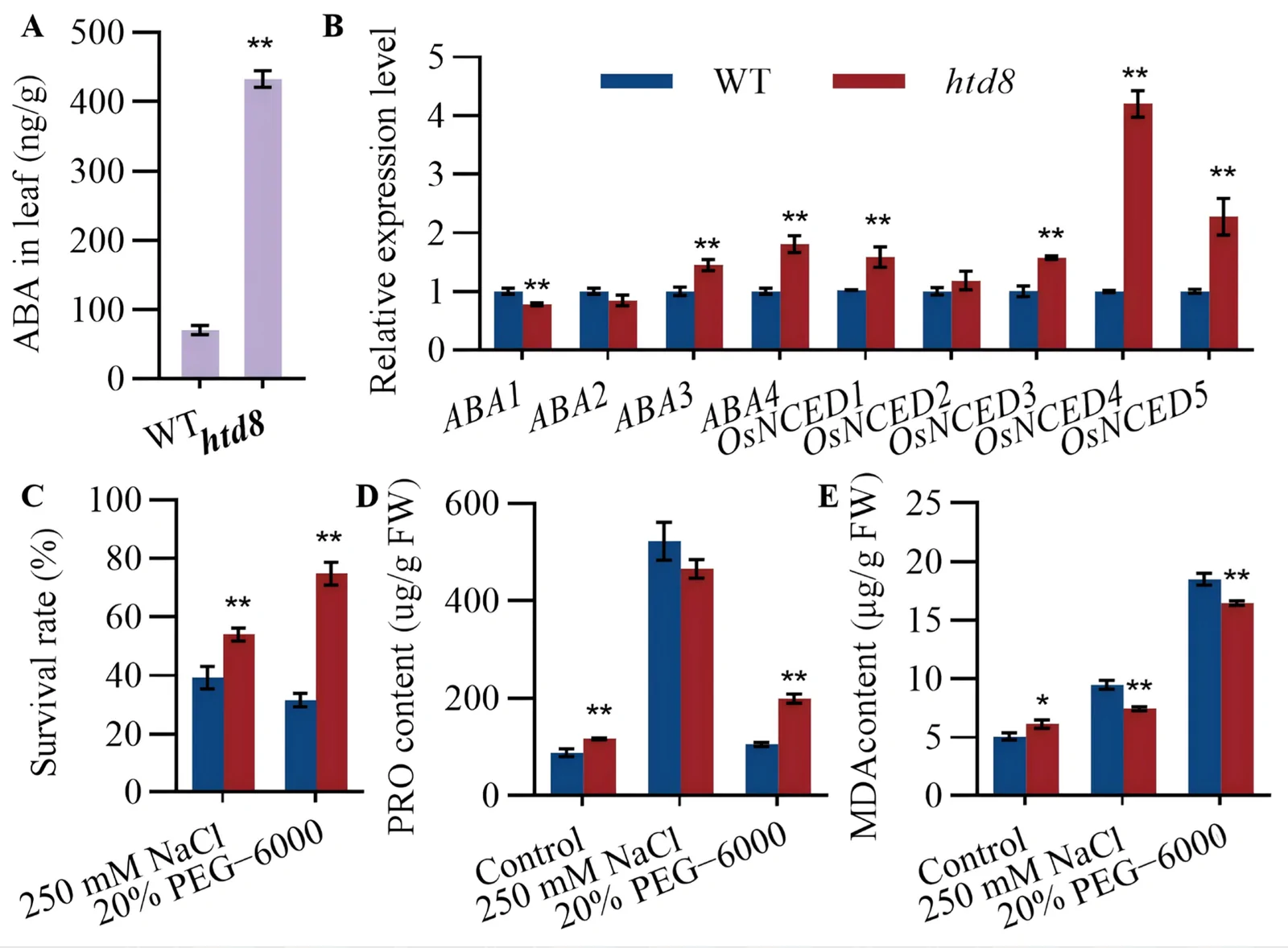
Abiotic stress responses and expression of stress-related genes in wild-type and htd8 mutant seedlings. (**A**) ABA content in leaves. (**B**) Expression analysis of ABA biosynthesis-related genes. (**C**) Survival rate after different stress treatments. (**D**) Proline content in leaves following abiotic stress. (**E**) MDA content in leaves following abiotic stress. * *p* < 0.05; ** *p* < 0.01.

Physiological measurements (Figure 6) revealed that proline accumulation in *htd8* was significantly higher than in the WT under PEG-induced osmotic stress, indicating enhanced osmotic adjustment capacity. Additionally, MDA content in *htd8* was significantly lower than in the WT under both salt and PEG-induced osmotic stress, suggesting reduced oxidative damage to cell membranes, improved membrane stability, and greater stress tolerance. Further analysis showed that ABA levels in the leaves of the mutant were much higher than those in the WT prior to stress treatment, and the expression levels of ABA biosynthesis-related genes were also significantly upregulated, consistent with the observed ABA accumulation pattern. Based on these findings, we propose that *HTD8* may mediate plant responses to salt and PEG-induced osmotic stress by regulating the ABA biosynthesis pathway.

### 2.5. Genetic Analysis of the htd8 Mutant

After crossing the *htd8* mutant with ZH11, the F_1_ progeny exhibited normal phenotypes, indicating that the mutant trait is controlled by a recessive locus. As shown in Table 1, selfing of the F_1_ population yielded an F_2_ generation with phenotypic segregation consisting of 121 normal plants and 28 plants exhibiting the *htd8* mutant phenotype, corresponding to a segregation ratio of 4.32:1. Chi-square analysis revealed that this ratio fits the expected 3:1 ratio (χ^2^ = 0.38 < χ^2^_0.05_), consistent with Mendel’s law of gene segregation, confirming that the *htd8* trait is governed by a single recessive nuclear gene.

### 2.6. Positioning of htd8

**Figure 7:**
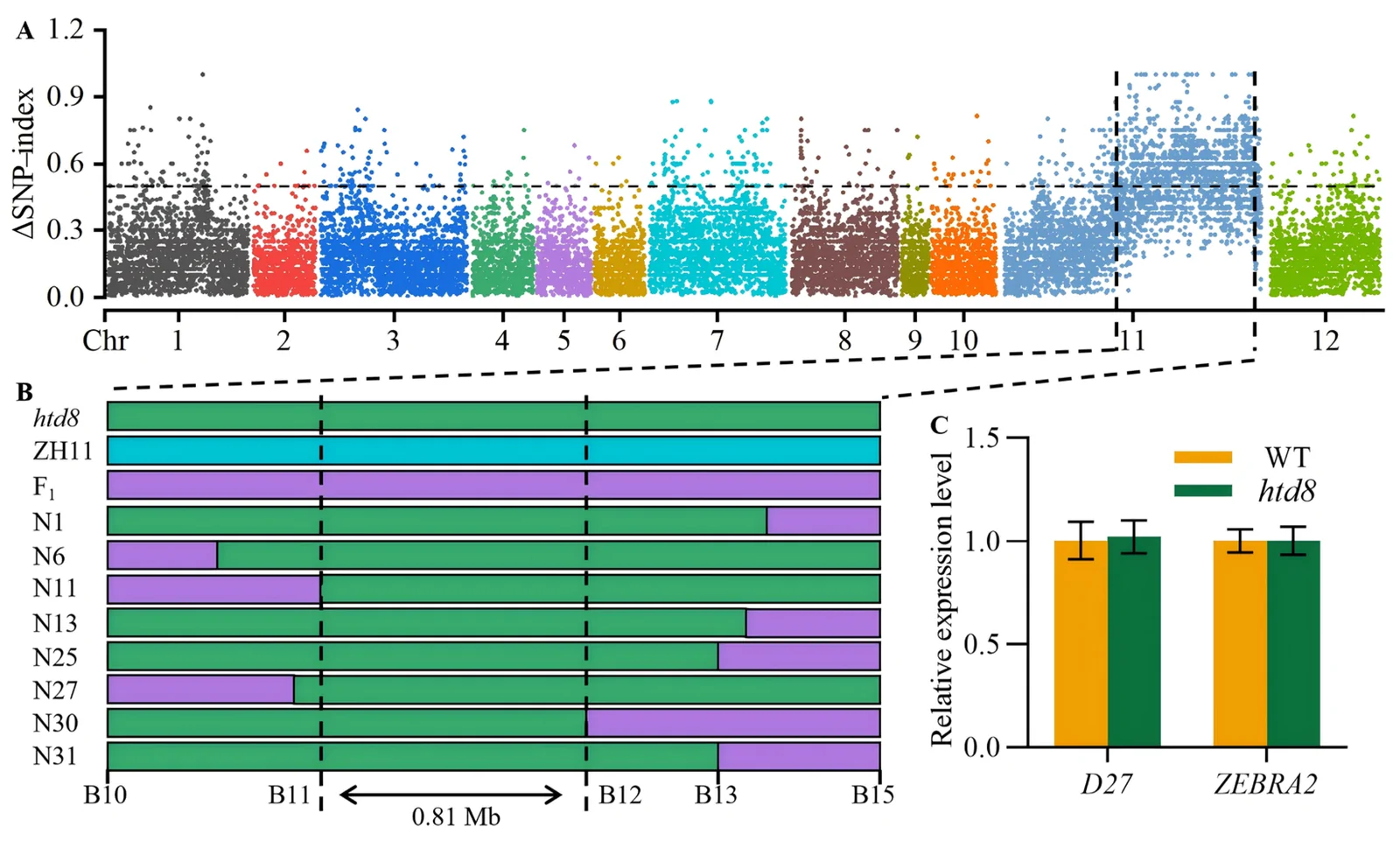
Genetic mapping of the rice *HTD8* gene. (**A**) Manhattan plot fitted by the index-slid method; each color corresponds to one chromosome. (**B**) Fine mapping of the *HTD8* gene, with major InDel markers labeled. The *HTD8* gene is narrowed down to the region between *B10* and *B15*. Green indicates the *HTD8* genomic region, blue represents the ZH11 genomic region, and purple denotes heterozygous regions. (**C**) Expression analysis of *D27* and *ZEBRA2* in leaves of WT and mutant plants at the early tillering stage.

To determine the chromosomal location of the *htd8* mutant gene, an F_2_ population was constructed by crossing the *htd8* mutant with TN1. Genomic sequencing and Bulk Segregant Analysis (BSA) were performed on pooled DNA samples from both parents and two progeny generations. The results revealed a major effect peak on chromosome 11, located between 22 369 198 and 25 635 987 bp. To further narrow down the position of the *htd8* gene, five InDel markers were synthesized, reducing the candidate region to 0.81 Mb. Sequencing and quantitative analysis of the genes *D27* and *ZEBRA2* within this interval showed no mutations in either (Figure 7).

### 2.7. Transcriptome Functional Enrichment Analysis

**Figure 8:**
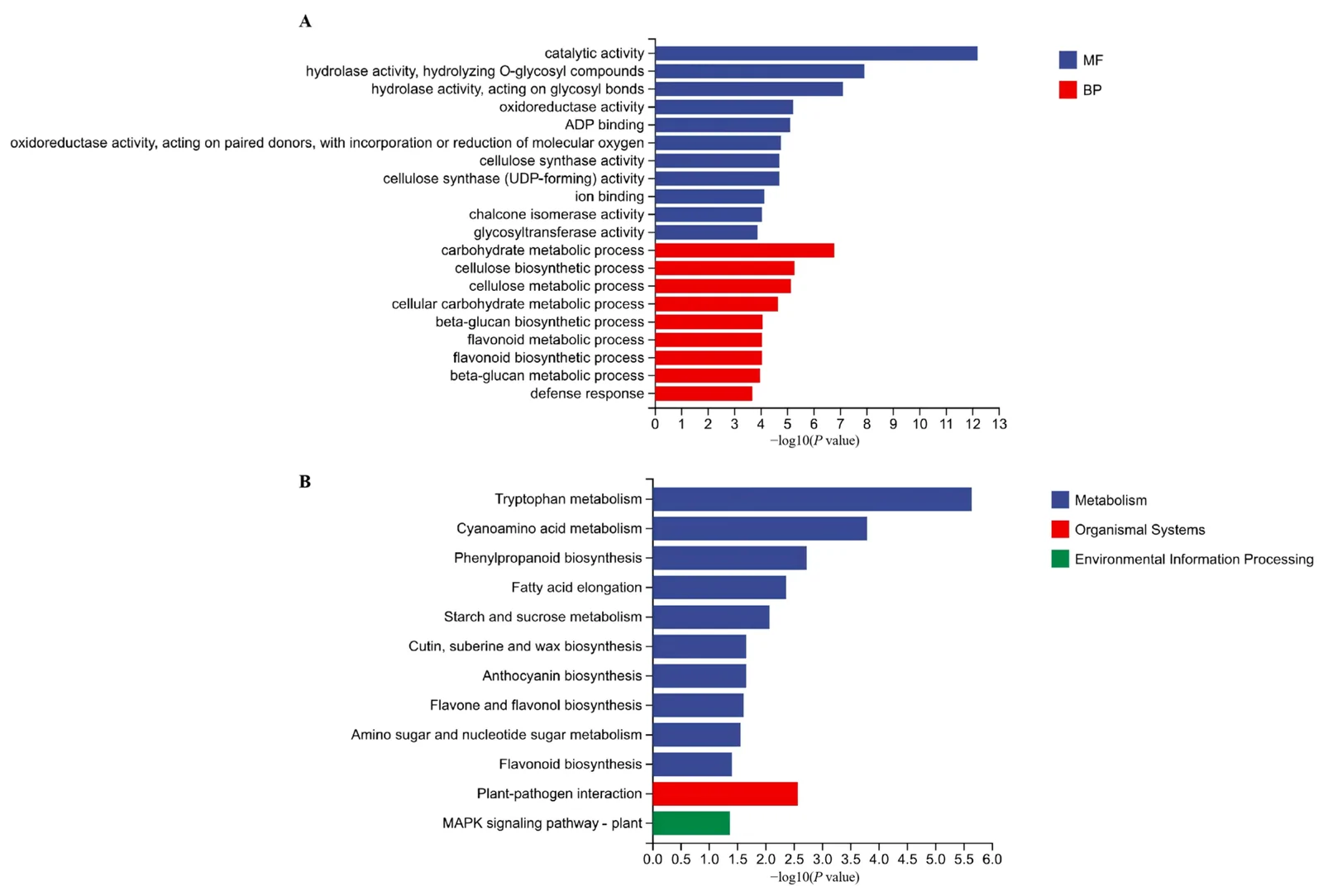
The results of transcriptome functional enrichment analysis. (**A**) The results of GO enrichment analysis. (**B**) The results of KEGG enrichment analysis.

To dissect the mechanism underlying the high-tillering phenotype of the *htd8* mutant, tiller bud samples were collected at the early tillering stage for transcriptome analysis. GO enrichment analysis (Figure 8A) demonstrated that DEGs were enriched in molecular function (MF) categories: catalytic activity (GO: 0003824), hydrolase activity acting on glycosyl bonds (GO: 0016798), oxidoreductase activity (GO: 0016491), and cellulose synthase activity (GO: 0016759); and biological process (BP) categories: carbohydrate metabolic process (GO: 0005975), cellulose biosynthetic process (GO: 0030244), flavonoid biosynthetic process (GO: 0009813), and defense response (GO: 0006952). KEGG enrichment (Figure 8B) indicated that these DEGs were significantly enriched in metabolic pathways, including tryptophan metabolism, phenylpropanoid biosynthesis, anthocyanin biosynthesis, starch and sucrose metabolism, as well as environmental response pathways including the MAPK signaling pathway and plant–pathogen interaction. The significant enrichment of the tryptophan metabolism pathway suggests obvious perturbation in tryptophan-mediated auxin-related metabolic processes in the mutant. This may alter the dormancy and outgrowth status of lateral buds by modulating apical dominance, thereby promoting tiller formation. The enrichment of phenylpropanoid and anthocyanin biosynthesis pathways reflects remodeling of the secondary metabolic network. The redistribution of carbon metabolic flux provides material support for the growth and development of tiller buds. Furthermore, enrichment of fatty acid metabolism-related pathways implies that the metabolism of strigolactone precursors is disturbed, which participates in the regulation of tiller bud growth. Collectively, the coordinated perturbation of these GO functional terms and KEGG pathways mediates the high-tillering phenotype of the *htd8* mutant.

## 3. Discussion

Rice is one of the most important food crops globally, and continuously improving its yield and adaptability remains a central focus in crop science research [23]. Tillering number and plant height are key agronomic traits determining rice plant architecture and yield. Although numerous genes regulating tiller initiation and elongation have been cloned to date [24], few pleiotropic genes capable of simultaneously coordinating plant architecture development, chloroplast formation, and abiotic stress responses have been identified [25]. Therefore, cloning and comprehensive characterization of key genes controlling tillering, hormone signaling, and stress response are of significant theoretical and practical importance for breeding high-yielding and stress-tolerant rice varieties [10]. The *htd8* mutant exhibits strong pleiotropic phenotypes including excessive tillering, dwarfism, abnormal chloroplast development, and significantly enhanced tolerance to salt and PEG-induced osmotic stress, providing an ideal experimental system for dissecting these complex traits.

In this study, the *htd8* mutant was obtained through EMS mutagenesis of the rice variety ‘SKZ’. The mutant displays a typical phenotype of multiple tillers and reduced plant height due to significantly shortened internodes between the first and third nodes (Figure 1C), while the number of tillers at maturity reaches approximately three times that of the WT (Figure 1A). Grain size is reduced, and thousand-grain weight is markedly decreased (Figure 1E, Table 1). Exogenous hormone treatments revealed that the aerial parts of *htd8* are insensitive to GA_3_ (Figure 4A–C) but show significantly enhanced sensitivity to the SL analog GR24 (Figure 4D–F). Both GA_3_ and GR24 suppress tiller formation (Figure 4G,H). Under salt stress (250 mmol·L^−1^ NaCl) and PEG-induced osmotic stress (20% PEG-6000) (Figure 5), *htd8* seedlings exhibit higher survival rates than the WT (Figure 6C), with even greater advantages under PEG-induced osmotic stress, accompanied by significantly elevated proline accumulation (Figure 6D), reduced MDA content (Figure 6E), and upregulated expression of ABA-related genes along with increased ABA levels in leaves (Figure 6A,B). Genetic analysis indicates that *htd8* is controlled by a single recessive nuclear gene. BSA sequencing combined with fine mapping narrows *HTD8* to a 0.81 Mb region on chromosome 11. Sequencing and expression analysis (Figure 7C) exclude known tiller-promoting genes *D27* and *ZEBRA2*. GO analysis (Figure 8A) and KEGG enrichment analysis (Figure 8B) further reveal coordinated perturbations in brassinosteroid, auxin, gibberellin, and strigolactone signaling pathways as well as carbon metabolism.

The increased tillering and reduced plant height observed in *htd8* closely resemble those seen in a series of SL-related high-tiller dwarf mutants in rice. Tillering in rice initiates via axillary meristem activation mediated by *MOC1* [6] and is suppressed by negative regulators such as *OsTB1*. Loss-of-function mutations in SL biosynthesis genes *HTD1*/*D17*, *D10*, *D27*, or the SL receptor *D14* all lead to increased tillering and reduced plant height [26,27,28,29]. Upon recognition by the receptor, SL promotes degradation of *D53*, thereby relieving its repression on tillering [30,31]. However, *htd8* shows hypersensitivity to GR24, and GR24 inhibits tillering in *htd8*, suggesting that the increased tillering is not simply due to SL deficiency but may reflect a coupling between GA and SL signaling. Similarly, in rice, GA-triggered degradation of the *DELLA* protein *SLR1* reduces the stability of the tiller activator *MOC1*, whereas SL acts through the *D14*-OsMADS23 module to counteract this process, enabling convergence of the two hormonal pathways in tiller regulation [12,18]. From a breeding perspective, the allelic form *HTD1^HZ^* of SL synthesis genes has been shown to increase tillering and improve yield without affecting other agronomic traits such as seed set rate, further highlighting the application value of this class of hub genes [22].

ζ-Carotene isomerase (Z-ISO) is also associated with rice tillering; its functional loss reduces the levels of carotenoid-derived hormones, impairs chloroplast development and photosynthesis, and increases tillering by lowering SL levels [32], while SL exhibits an antagonistic effect with on ABA [33]. In this study, *htd8* showed abnormalities in chloroplast ultrastructure (Figure 2G,H) and leaf phenotypes including reduced chlorophyll and carotenoid contents (Figure 3C), yet ABA levels were significantly elevated in htd8 (Figure 6A). This discrepancy suggests that *HTD8* may act at a branching point in the carotenoid pathway where precursors are diverted toward ABA synthesis rather than globally suppressing the entire pathway. Under salt and PEG-induced osmotic stress, *htd8* exhibited higher survival rates (Figure 6C), increased Pro accumulation (Figure 6D), reduced MDA levels (Figure 6E), and enhanced ABA synthesis (Figure 6A). The 9-*cis*-epoxycarotenoid dioxygenase (NCED) enzyme is the rate-limiting step in ABA biosynthesis. SL induces the expression of the transcription factor *OsHOX12*, which in turn regulates OsNCED1 to promote ABA synthesis. Liu et al. found that the synthetic SL analog rac-GR24 significantly induced *OsNCED1* expression in the base of rice stems, but this induction was nearly absent in *OsHOX12* knockout mutants, confirming that *OsHOX12* is essential for SL activation of NCED. Moreover, this pathway shows spatial specificity, primarily functioning in the stem base, thus explaining how SL suppresses lateral bud elongation by promoting ABA synthesis in the stem base [25]. NCED is strongly induced by NaCl and PEG, and its overexpression enhances rice tolerance to multiple abiotic stresses [34], which is partially reflected in this experiment (Figure 5). Pro accumulates extensively under stress as an osmoprotectant [35], while the significant reduction in stomatal density helps conserve water and enhance drought avoidance ability [36], contributing to the high survival rate of *htd8* under PEG-induced osmotic stress.

Transcriptome analysis revealed (Figure 8) significant enrichment of pathways related to phenylpropanoid biosynthesis, anthocyanin biosynthesis, and starch and sucrose metabolism in *htd8*, indicating a reprogramming of carbon metabolism and secondary metabolism allocation in the mutant. The enrichment in starch and sucrose metabolism involves multiple aspects including sugar supply, sucrose turnover, starch mobilization, and sugar utilization, possibly reflecting changes in sugar availability or metabolic status around tiller buds. Enrichment in phenylpropanoid and anthocyanin biosynthesis pathways may indicate a shift in carbon flux from primary metabolism toward secondary metabolism centered on the phenylpropanoid pathway. SL can inhibit the expression and polar localization of monosaccharide transporters *OsMST3*/*6*, thereby restricting glucose and fructose uptake by tiller buds and suppressing bud elongation [37]; it can also regulate whole-plant sugar distribution via the *OsSWEET16* pathway and interact with trehalose-6-phosphate signaling to suppress branching. Conversely, sucrose can counteract SL’s inhibition of rice tillering and promote *D53* protein accumulation [38]. These findings collectively suggest that changes in carbon allocation may partially contribute to the increased tillering observed in *htd8*. However, transcript abundance alone cannot reliably indicate the flux status of central metabolism, and pathway enrichment does not necessarily equate to a directed increase in carbon allocation toward tiller buds. Nevertheless, this hypothesis still requires direct metabolomic evidence for validation.

This study has several limitations. First, salt stress simultaneously induces osmotic and ionic toxicity; thus, we did not rigorously determine the dominant factor, as sodium and chloride ion uptake was not measured. Second, due to experimental constraints, PSII activity was not assessed to further investigate its impact on photosynthesis. Finally, *HTD8* has not yet been cloned, so the observed transcriptome changes remain correlative evidence, necessitating further investigation using mutants. These represent key directions for future research.

In summary, this study identifies *HTD8* as a novel locus involved in plant architecture development, chloroplast biogenesis, and ABA-mediated stress responses, characterized by pleiotropic effects and enhanced stress tolerance. Its properties make HTD8 a promising genetic resource for developing rice varieties with both high-yielding architecture and improved salt and drought resistance, offering a new avenue for sustainable food production through optimized tillering [2,39].

## 4. Materials and Methods

### 4.1. Experimental Materials and Agronomic Traits Evaluation

The rice multi-tillering dwarf mutant htd8 originated from an EMS mutagenized population of the indica variety ‘SKZ’, and the lesion trait was stably inherited after several generations of selfing. Using *htd8* as the female parent and japonica ZH11 as the male parent, artificial hybridization was performed; the spikelets of *htd8* were emasculated and pollinated with ZH11 pollen, then bagged to isolate and harvest F_1_ seeds.

F_1_ seeds were planted in winter at a trial base in Hainan, with single plants transplanted at a spacing of 20 cm × 25 cm. The parents *htd8* and ZH11 served as controls, and field management was conducted conventionally. Phenotypes of F_1_ plants were evaluated, and false hybrids were removed. Each F_1_ plant was selfed, and F_2_ seeds were harvested separately for each individual. The following year, F_2_ seeds were raised as seedlings in Hangzhou, transplanted after 30 days of seedling growth, planted individually at 20 cm × 25 cm spacing, with protective rows established. A total of 149 F_2_ plants were used for genetic segregation analysis and gene mapping; extreme individuals showing multi-tillering and dwarfism were selected for leaf sampling, which were used for BSA sequencing and fine mapping [40].

### 4.2. Leaf Observation by Scanning Electron Microscopy and Transmission Electron Microscopy

WT ‘SKZ’ and mutant *htd8* mature leaves without veins were selected, cut into 1 × 3 mm fragments, and immersed in 2.5% glutaraldehyde solution under vacuum for 1 h, then fixed overnight at 4 °C. The samples were rinsed three times with pH 7.0, 0.1 M phosphate-buffered saline, each for 15 min. They were subsequently dehydrated through a graded ethanol series, followed by final dehydration in absolute ethanol, critical-point dried using a critical point dryer, coated with metal, and observed and imaged via scanning electron microscopy (Thermo Fisher Scientific, Waltham, MA, USA).

Sample initial fixation and buffer rinsing procedures were identical to those used in scanning electron microscopy sample preparation. Samples were transferred into 1% osmium tetroxide solution for 1–2 h of fixation, followed by thorough rinsing. Dehydration was carried out gradually using ethanol and acetone, followed by stepwise infiltration with Spurr embedding medium mixed with acetone at different ratios, and finally left in pure embedding medium overnight. After paraffin embedding, the samples were heated and cured at 70 °C. Ultrathin sections (70–90 nm) were cut using an ultramicrotome, stained with double staining, and examined and photographed under transmission electron microscopy (Thermo Fisher Scientific, Waltham, MA, USA) [41].

### 4.3. Determination of Photosynthetic Pigment Content

Leaf samples (0.2–0.3 g) collected from the same position were immersed in 5 mL of 95% ethanol and vacuum-infiltrated for 30 min to ensure complete immersion in the ethanol solution. Extraction was performed at 4 °C in darkness for 24 h with periodic shaking until the pigments were completely extracted. The absorbance of the supernatant was measured at 470, 649, and 665 nm using a Tecan Infinite M Nano microplate reader (Tecan Austria GmbH, Grödig, Austria). Contents of chlorophyll a (Chl a), chlorophyll b (Chl b), and carotenoids (Car) were calculated according to the Lichtenthaler method [42].

### 4.4. Exogenous Plant Hormone Treatment of Seedlings

Mature seeds of WT and *htd8* mutant were mechanically dehulled, disinfected with 70% ethanol for 2 min, followed by treatment with 30% sodium hypochlorite solution for 30 min. After disinfection, the seeds were rinsed with sterile water and inoculated onto 1/2 MS medium supplemented with different concentrations and combinations of plant hormones. The seeds were cultured in an artificial climate incubator, after which root length and plant height were measured and photographs were taken. The incubator was set to a 14 h light/10 h dark photoperiod at 28 °C [43].

### 4.5. Abiotic Stress Effects on htd8

Healthy seedlings of WT and *htd8*, grown for 14 days, were selected and subjected to salt stress (250 mM NaCl) and osmotic stress induced by PEG (20% PEG-6000), respectively. After clear phenotypic differences emerged, the seedlings were transferred back to normal nutrient solution for recovery. Seedling survival was determined as follows: seedlings were considered alive if they produced new green leaves and continued to grow after 7 days of recovery, while those with completely yellowed and necrotic leaves and no new tissue formation were considered dead. The survival rate of WT and *htd8* seedlings was calculated. Additionally, expression levels of stress-responsive genes and *HTD8* itself were analyzed before and after stress treatment [44,45].

Survival rate = (number of surviving plants/total number of treated plants) × 100%

### 4.6. Abscisic Acid Content Determination

Fresh and tender rice leaves were ground to a fine powder in liquid nitrogen. A 200 mg sample was weighed into a glass tube, and 10 volumes of acetonitrile were added. The mixture was extracted overnight at 4 °C and then centrifuged at 12,000× *g* for 5 min. The supernatant was collected, and the pellet was re-extracted twice with 5 volumes of acetonitrile. The resulting supernatants were combined, and 200 mg of C18 sorbent was added. The mixture was vortexed vigorously for 30 s and centrifuged at 10,000× *g* for 5 min. The supernatant was collected, evaporated to dryness under a nitrogen stream, and reconstituted in 150 μL of methanol. The reconstituted solution was filtered through a 0.22 μm organic-phase membrane and stored at −20 °C. The liquid chromatography detection method was performed as described by Hu et al. [46].

### 4.7. Measurement of Antioxidant-Related Physiological Parameters

Leaves were collected before and after stress treatment as samples for physiological index measurements. The experiment was divided into control and experimental groups, each with three biological replicates. Procedures were performed according to the instructions of the Proline (Pro) and MDA content detection kits (Solarbio Science & Technology Co., Ltd., Beijing, China) [47,48,49].

### 4.8. RNA Extraction and Real-Time Quantitative Polymerase Chain Reaction (qRT-PCR) Detection

Total RNA was extracted from WT and htd8 mutant plants using an RNA extraction kit (UElandy, Shanghai, China) and reverse-transcribed into cDNA. Rice *OsActin* was used as the internal control gene for qRT-PCR. The reaction mixture (10 μL) consisted of 1 μL cDNA template (500 ng·μL^−1^), 1 μL each of forward and reverse primers (10 μmol·L^−1^), 5 μL SYBR Green Realtime PCR Master Mix, and 2 μL RNase-free water. Each reaction was performed in triplicate (one internal reference per cDNA template). The amplification program included: 95 °C for 5 min, followed by 40 cycles of 95 °C for 10 s, 57 °C for 20 s, and 72 °C for 20 s. Data were analyzed using the 2^−ΔΔCt^ method [50].

### 4.9. Genetic Analysis and Mapping

Extreme phenotype individuals from the *htd8* × ZH11 F_2_ segregating population were selected to construct DNA pools for Bulk Segregant Analysis (BSA). The sequencing libraries were prepared and sequenced by Shanghai Majorbio Bio-Pharm Technology Co., Ltd., Shanghai, China (https://www.majorbio.com/). Following sequence alignment and variant calling, candidate genomic intervals were identified based on SNP-index association analysis. Genomic DNA was extracted using the Tissue Preparation Solution (TPS) method. Fine mapping was performed using InDel markers developed in this study. PCR amplification was conducted with a reaction volume of 10 μL, containing 2 μL template DNA, 2 μL ddH_2_O, 4 μL 2 × Taq Mix, and 1 μL each of forward and reverse primers (Table 2). The thermal cycling conditions were as follows: initial denaturation at 95 °C for 5 min; 35 cycles of denaturation at 95 °C for 15 s, annealing at 55 °C for 15 s, and extension at 72 °C for 30 s; followed by the final extension at 72 °C for 5 min and storage at 16 °C for 10 min. PCR products were separated by electrophoresis on a 4% agarose gel. A physical map was constructed using rice genome annotation data retrieved from the Gramene database (http://www.gramene.org) [51].

### 4.10. Transcriptome Sequencing Analysis

Total RNA was extracted from tiller buds at the early tillering stage, and samples were sent to Personal Biotechnology, Shanghai, China. (http://www.personalbio.cn/) for transcriptome sequencing (BioProject accession SUB16570609). After data alignment and expression level analysis, differentially expressed genes were identified, followed by functional annotation and enrichment analysis to elucidate the molecular regulatory mechanisms associated with the trait [52].

## 5. Conclusions

In conclusion, we identified a novel rice recessive mutant *htd8* from the EMS-mutagenized population of cultivar ‘SKZ’. The *htd8* mutant displays multiple pleiotropic phenotypes including dwarfism, excessive tillering, narrow chlorotic leaves, impaired photosynthesis and reduced grain yield. The mutation alters plant hormone responses: *htd8* is insensitive to GA_3_ but hypersensitive to the strigolactone analog GR24, and the GA and SL signaling pathways cooperatively regulate tiller development. Meanwhile, the *htd8* mutant exhibits enhanced tolerance to salt and osmotic stress, which is attributed to activated ABA biosynthesis, improved osmotic adjustment, and stabilized cell membrane stability. Genetic mapping localized *HTD8* to a 0.81 Mb interval on chromosome 11, distinct from previously reported tillering-related genes, indicating that *HTD8* represents an uncloned novel gene. Transcriptome evidence demonstrates that the combined perturbation of multiple phytohormone pathways and redistribution of carbon metabolic flux underlie the phenotypic formation of *htd8*. Collectively, our findings reveal that *HTD8* plays an essential role in regulating rice plant architecture, hormone homeostasis and abiotic stress adaptation. This work provides important genetic material and theoretical basis for molecular breeding of high-yield and stress-tolerant rice.

## Figures and Tables

**Table 1:** Genetic analysis of the trait.

Crossingcombination	Normalphenotype	Mutantphenotype	Summation	χ^2^_0.05_
*htd8*/ZH11	121	28	149	0.38

**Table 2:** Primer sequences.

Primername	Forwardprimer (5′–3′)	Reverseprimer (5′–3′)	Purpose
B10	CGGCATGTCATGGACTACG	CAGGAAATCTGTAACCAGAGG	Mapping
B11	TCAAATTTCCCATGCCACCAA	CTCACGGCTCACGCCAACCCA	
B12	TGAACCCTGCTCTTCTGAGTC	AAAGAAGATATGAAGGCACCG	
B13	CTTCATAAATGATACATGGTGTC	TTTTGGGATTGTCCTTCTTCG	
B15	ACAGCTATTGAATCGGGAC	CCTAATACCAACACTACTGGAG	
*OsActin*	TGGCATCTCTCAGCACATTCC	TGCACAATGGATGGGTCAGA	qRT-PCR
ABA1	GGATGCCATTGAGTTTGGTT	TGGCTGACTGAAGTCTCTCG	
ABA2	AGCAAACCTGAAAGGTGTGGA	AAAGCCACCATCCACCATGA	
ABA3	GGGCAAGATTTTGTTCGGCA	AAGGGTACACTTGTTGCCCC	
ABA4	CTCTGTTCCGGCCTCGCGCA	TCAATGTGAGCGACCAATTGAAC	
OsNCED1	TGTTATTGCTACGACGAATGGC	GGCGGGTCGATGAACGTG	
OsNCED2	GGTATGGAAACGAGGATAGTGGTT	TGCTTATTGTTGTGCGAGAAGTTC	
OsNCED3	CTCCCAAACCATCCAAACCG	TGAGCATATCCTGGCGTCGT	
OsNCED4	CCCTCAGCTACGACGTGATC	AACATCTCGCCGAGCTTGAA	
OsNCED5	CCCTCAGCTACGACGTGATC	AACATCTCGCCGAGCTTGAA	
D27	TGCTGCATTCACCACGATA	ACCAACACAATTTGTACTTTCCA	
ZEBRA2	GGGAACTTGAATTTGATTACACAAG	GCGTCAAACATTCCAGAGGC	

## Data Availability

The original contributions presented in this study are included in the article/Supplementary Materials. The transcriptome sequencing data generated in this study have been deposited in the NCBI Sequence Read Archive (SRA) and are accessible under the BioProject accession SUB16570609. Further inquiries can be directed to the corresponding author.

## Supplementary Materials

The following supporting information can be downloaded at: https://www.mdpi.com/article/10.3390/plants15213335/s1, Table S1: BSA-index.
